# Long-Term Neuropsychological Sequelae in HIV-Seronegative Cryptococcal Meningoencephalitis Patients with and without Ventriculoperitoneal Shunts: A Cine MRI Study

**DOI:** 10.1155/2015/356476

**Published:** 2015-04-09

**Authors:** Meng-Hsiang Chen, Cheng-Hsien Lu, Hung-Chen Wang, Hsiu-Ling Chen, Nai-Wen Tsai, Shau-Hsuan Li, Nai-Wen Hsu, Wei-Ming Lin, Chia-Te Kung, Wei-Che Lin

**Affiliations:** ^1^Department of Diagnostic Radiology, Kaohsiung Chang Gung Memorial Hospital, Chang Gung University College of Medicine, Kaohsiung 83305, Taiwan; ^2^Department of Neurology, Kaohsiung Chang Gung Memorial Hospital, Chang Gung University College of Medicine, Kaohsiung 83305, Taiwan; ^3^Department of Biological Science, National Sun Yat-Sen University, Kaohsiung 804, Taiwan; ^4^Department of Neurosurgery, Kaohsiung Chang Gung Memorial Hospital, Chang Gung University College of Medicine, Kaohsiung 83305, Taiwan; ^5^Department of Biomedical Imaging and Radiological Sciences, National Yang-Ming University, Taipei 11221, Taiwan; ^6^Department of Medicine, Kaohsiung Chang Gung Memorial Hospital, Chang Gung University College of Medicine, Kaohsiung 83305, Taiwan; ^7^Department of Radiology, Yuan's General Hospital, Kaohsiung 802, Taiwan; ^8^Department of Radiology, Chiayi Chang Gung Memorial Hospital, Chiayi 613, Taiwan; ^9^Department of Emergency Medicine, Kaohsiung Chang Gung Memorial Hospital, Chang Gung University College of Medicine, Kaohsiung 83305, Taiwan

## Abstract

*Background.* Hydrocephalus in cryptococcal meningoencephalitis is most commonly managed with a ventriculoperitoneal shunt. This study applied cine magnetic resonance imaging (MRI) to evaluate initial disease severity on long-term cerebrospinal fluid (CSF) flow dynamics and associated neuropsychological sequelae in cryptococcal meningoencephalitis patients with and without ventriculoperitoneal shunts. *Methods.* Eighteen human immunodeficiency virus-seronegative cryptococcal meningoencephalitis patients (10 with shunts versus 8 without shunts) were compared with 32 age- and sex-matched healthy volunteers. All subjects underwent complete neurologic examination and neuropsychological testing. Cine MRI was conducted to evaluate CSF flow parameters. Initial CSF laboratory analysis and imaging findings were correlated with present CSF flow parameters and neuropsychological scores. *Results.* Patients without shunts had higher average flow than controls, suggesting chronic hydrocephalus. Initial Evans ratios and CSF glucose levels were associated with CSF peak velocity and flow. Worsening CSF flow parameters correlated with decreased neuropsychological performance. *Conclusions.* CSF flow parameter differences between the cryptococcal meningoencephalitis patients both with and without ventriculoperitoneal shunts could be detected by cine MRI and correlated with acute stage disease severity and chronic stage neuropsychological results. Cine MRI is useful for assessing the chronic hydrocephalus that may lead to neuropsychological deficits in cryptococcal meningoencephalitis patients.

## 1. Background

Cryptococcal meningoencephalitis is the most frequently encountered manifestation of cryptococcal infection. It has a high rate of neurologic sequelae despite antifungal therapy [1–3]. Hydrocephalus is an occasional complication. Delays in hydrocephalus diagnosis and treatment are directly related to poor outcomes, including various degrees of residual neurologic sequelae [4–7].

Ventriculoperitoneal shunt placement is a helpful intervention to avoid fatal acute stage complications caused by hydrocephalus. However, shunt placement for hydrocephalus does not always result in a good outcome [5, 6]. Cryptococcal meningoencephalitis patients without hydrocephalus do not initially require a shunt procedure. However, chronic reinfection or delayed increases of intracranial pressure may occur and lead to clinical deterioration. The long-term dynamics of cerebrospinal fluid (CSF) in cryptococcal meningoencephalitis are unclear, and the effects of cryptococcal meningoencephalitis on long-term neuropsychological sequelae likewise remain unknown. Improved treatment strategies may help, however, to increase the understanding of cognitive impairment that occurs in such patients.

Cine magnetic resonance imaging (MRI) has been applied to a variety of clinical uses [7] because of its ability to noninvasively evaluate flow characteristics inside the body. It can help determine the possible pathophysiology of normal pressure hydrocephalus using peak velocity and flow parameters. The current study primarily sought to test the hypothesis that changes in CSF dynamics occur in chronic cryptococcal meningoencephalitis patients during long-term follow-up irrespective of whether they receive a ventriculoperitoneal shunt. The study also used neuropsychological testing to examine the relationships among initial presentations, alterations in CSF dynamics, and cognitive function.

## 2. Methods

### 2.1. Subjects

In Taiwan, the majority of patients with cryptococcal meningitis are HIV-negative [8], although the condition is associated with diabetes mellitus (18.9%), liver cirrhosis (7%), and iatrogenic Cushing syndrome (7%) [9]. At Kaohsiung Chang Gung Memorial Hospital, there were 64 patients over a period of 13 years (1998–2010) who were retrospectively identified as having cryptococcal meningoencephalitis. Among these 64 patients, there were 21 patient deaths. In the group of 43 survivors, there were 25 patients who received regular follow-ups. All these patients were followed up at the hospital's neurology outpatient clinic for more than one year after complete antifungal therapy, and none of them had impairment of the body's immune system. The initial CSF laboratory presentations, acute stage MRI, and follow-up examinations (including brain cine MRI and neuropsychological tests) were then analyzed. The hospital's ethics committee approved this retrospective study (IRB 101-2469B).

Cryptococcal meningitis was diagnosed according to previously published data [1]. In addition, cryptococcal meningoencephalitis was defined as (1) isolation of* Cryptococcus neoformans* in one or more CSF cultures, positive CSF cryptococcal-antigen titer, or positive CSF India ink staining, along with clinical features of meningitis or (2) isolation of* C. neoformans* in blood culture with clinical presentations and typical CSF features of meningitis [10].

Cryptococcal meningoencephalitis patients were excluded if they had any of the following: (1) age of <20 years or >75 years; (2) evidence of alcoholism, known sedative or neuroleptic use related to affective or psychiatric disease or any other addictive disorders; (3) known neurologic disorders potentially affecting the central nervous system or severe recent life events possibly interfering with neuropsychological testing; (4) poor neuroimaging quality. Among the 25 patients, one was excluded due to age, three were excluded due to alcoholism, two were excluded due to medication for insomnia, and one was excluded due to poor imaging quality resulting from noncooperation during MR acquisition. As such, a final total of 18 cryptococcal meningoencephalitis patients were enrolled in this retrospective study. Two of them had diabetes mellitus, one had liver cirrhosis, and another had a history of idiopathic thrombocytopenia with steroid exposure. The 18 patients finally included were divided into two groups according to whether or not they received ventriculoperitoneal shunts.

All of the patients underwent complete medical and neurologic examinations, as well as neuropsychological testing. Neurologists integrated both the clinical manifestations and neuropsychological findings. A cohort of 32 sex- and age-matched healthy subjects was recruited to serve as the control group; none of these subjects had any neurological disease and all had similar educational statuses.

### 2.2. MRI Study

#### 2.2.1. Initial Assessment

Cranial CT scans and/or MRI studies were performed on the patients upon admission. Repeat CT and/or MRI scans were conducted if there was any clinical deterioration before discharge. The following imaging characteristics were evaluated: (1) meningeal/gyral enhancement, (2) basal ganglia infarction/Virchow-Robin space dilatation, (3) hydrocephalus, and (4) focal cerebritis. Hydrocephalus was diagnosed by the presence of a dilated temporal horn of the lateral ventricle, without obvious brain atrophy and/or an Evans ratio of >0.3 on CT or MRI at admission. The Evans ratio refers to the ratio of the bilateral frontal horn ventricular width to the maximum biparietal diameter [11]. Hydrocephalus patients received a ventriculoperitoneal shunt upon evidence of either increased intracranial pressure or clinical deterioration.

#### 2.2.2. Follow-Up Examination

A follow-up MRI was performed using a 3 T unit (Signa Excite HD 12.0 Twin Speed 8-channel scanner; GE Medical Systems, Milwaukee, WI, United States) with a maximum slew rate of 150 T/m/s and a maximum gradient amplitude in each orthogonal plane of 50 mT/m. Intracranial lesions were evaluated using axial T1-weighted images, T2-weighted images, and T1-weighted enhanced images.

CSF flow quantification was performed using a cine phase contrast velocity-encoded pulse-gated single slice gradient echo sequence with a TE/TR of 7.5/33 msec, a slice thickness of 5 mm, a velocity encoding of 20 cm/s, and 32 phases acquired corresponding to the cardiac cycle. Other relevant scan parameters included a 20° flip angle, an FOV of 10.0 cm, and a phase FOV of 100%. All subjects underwent MRI examination at approximately the same time of day (i.e., the afternoon) to control for circadian variations [12].

#### 2.2.3. Evaluation of Cine MRI

**Figure 1:**
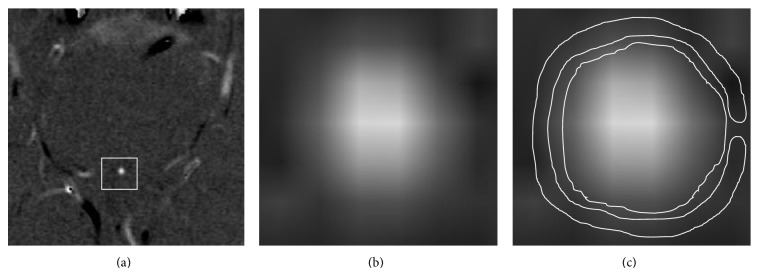
The original phase contrast imaging is shown in (a) and amplified in (b). The ROIs were placed manually to circulate through the whole aqueduct. The control reference of periaqueductal brain parenchymal matter is shown in (c) as a “C-shape” circulation area that avoided the basilar-vertebral vessel system.

The CSF flow data were processed using GE ReportCard software (version .3.0: General Electric Medical Systems, Waukesha, WI, United States), and all the parameters were recorded, including peak positive velocity (PPV), peak negative velocity (PNV), average flow (AF), average positive flow (APF), and average negative flow (ANF). Measurement biases were avoided through a discussion establishing a consensus among the authors. The ROIs were placed manually by two radiologists who were aided by a cursor and graphic display device; they covered the entire aqueduct, avoiding the brain parenchymal, CSF, and basilar-vertebral vessels, as demonstrated in Figure 1. The measurements were performed by investigators blinded to the patient's name, clinical findings, and other imaging data.

The mean values of the CSF flow parameters for each assessment were averaged from three repeated measurements. Intraobserver reproducibility (M.H.C.) was high for all the CSF flow parameters (1, 1, 0.927, 0.997, and 0.993 for PPV, PNV, AF, APF, and ANF, resp.). There was also high interobserver reproducibility (between M.H.C and W.C.L) for all CSF flow parameters (1, 1, 0.988, 0.998, and 0.994 for PPV, PNV, AF, APF, and ANF, resp.).

### 2.3. Neuropsychological Testing during Follow-Up

A clinical psychologist blinded to the patients' exposure status performed the neuropsychological tests. The patients completed a test battery which was assembled to assess executive function, attention, and memory. The neuropsychological evaluation was performed using Wechsler Adult Intelligence Scale-III subtests [13]. These included information measuring general knowledge, digit span, vocabulary (i.e., ability to define 35 words), arithmetic, letter-number sequencing, comprehension, similarities, picture completion, matrix reasoning, block design, picture arrangement, digit symbol, and object assembly.

The Cognitive Ability Screening Instrument [14] was obtained in all subjects for a brief screening cognitive assessment test comparing Asian and American populations. The test consists of nine domains of cognitive function (i.e., attention, concentration, orientation, short- and long-term memory, language ability, visual construction, word list generation, abstraction, and judgment), with scores ranging from 0 (worst) to 100 (best).

### 2.4. Statistical Analysis

The age and educational levels of the subjects in the three groups were analyzed using Student's *t*-test. Sex differences were analyzed by Fisher's exact test. Laboratory data that fit the Gaussian distribution were analyzed using the one-way analysis of covariance model with the patient's age, sex, and educational level as covariates. The laboratory data that did not have a Gaussian distribution were analyzed using the nonparametric independent samples test. The neuropsychological test scores between patients and control subjects were estimated by the one-way analysis of covariance model with the patient's age, sex, and education level as covariates.

Each of the CSF flow parameters for the three groups was estimated by one-way analysis of covariance models with the patient's age and sex as covariates; the Bonferroni correction was performed as a post hoc analysis. Secondary partial correlation analyses (two-tailed) were performed between CSF flow parameters and initial presentation in the acute stage and between CSF flow parameters and neuropsychological tests during follow-up; these tests were performed after controlling for age, sex, and educational level, demonstrating differences among the groups. Statistical significance was defined at *P* < 0.05. All statistical analyses were performed using SPSS software, version 17.0 (SPSS Inc., Chicago, IL, United States).

## 3. Results

### 3.1. Baseline Characteristics and Imaging Findings

At the time of admission, all 18 patients received complete laboratory tests and conventional CT and/or MRI studies to assess the disease. At the time of long-term follow-up, all 18 patients received complete neuropsychological testing, conventional MRI, and cine MRI for CSF flow parameter acquisition.

The baseline characteristics and initial clinical data of all subjects (i.e., patients and controls) are listed in Table 1. There were no significant differences among the three groups in terms of sex, age, or educational attainment. On average, the patients with shunts had a longer duration of hospitalization (*P* = 0.016), higher initial CSF lactate concentration (*P* = 0.028), and higher maximum CSF lactate concentration (*P* = 0.039) during the acute stage compared to patients without shunts.

Table 2 lists the acute stage neuroimaging findings and CSF cryptococcal-antigen titers on admission for each patient, along with their follow-up CT and/or MRI findings. Baseline studies were normal in five of the patients, all of whom were in the patient group without shunts.

During follow-up, the evolution of interval imaging studies was compared lesion by lesion. There was imaging abnormality resolution in nine of 18 patients (50%), including three patients (30%) in the shunt group and six patients (75%) in the group without shunts. There was a persistent abnormality in nine (50%) patients as follows: seven were in the patients with shunts (70%) group and two were in the patients without shunts (25%) group. Among the patients with shunts, five had parenchymal hyperintensity (50%), four had meningeal/gyral enhancement (40%), two had ventricular dilatation (20%), and one had Virchow-Robin space dilatation (10%). There were two patients with persistent abnormalities who had ventricular dilatation (25%) in the group without shunts.

### 3.2. Neuropsychological Testing

Patient neuropsychological tests were performed in the chronic disease stage, with the results compared with corresponding results for the controls. All the data are presented in Table 3. Each cryptococcal meningoencephalitis patient scored lower on the vocabulary, similarity, comprehension, digit span, picture complete, block design, and digit symbol coding tests of the Wechsler Adult Intelligence Scale-III than the healthy subjects did. The cryptococcal meningoencephalitis patients also scored more poorly on the orientation, abstract thinking, mental manipulation, short-term memory, and semantic fluency tests of the Cognitive Ability Screening Instrument than the healthy subjects did.

For most items, the patients with shunts received poorer neuropsychological scores than both the patients without shunts and the control subjects. Through one-way analysis of covariance after adjustments for age, sex, and education, the patients with shunts showed significantly poorer vocabulary, similarity, and digit symbol coding test results for the Wechsler Adult Intelligence Scale-III, as well as poorer attention test results for the Cognitive Ability Screening Instrument, than the controls.

However, the patients without shunts exhibited significantly lower block design results for the Wechsler Adult Intelligence Scale-III, as well as lower orientation and short-term memory results for the Cognitive Ability Screening Instrument, than the controls. Moreover, these results were even lower than those for the patients who had shunts.

### 3.3. CSF Flow Parameters

**Figure 2:**
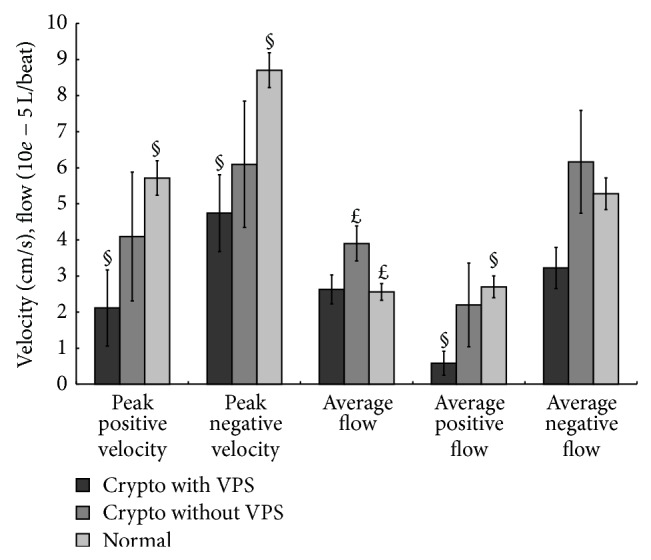
Cine MRI parameters of patients with and without shunts as compared with healthy controls. ^§^Significant difference between the patients with shunts and controls. ^*£*^Significant difference between the patients without shunts and controls (*P* < 0.05).

The patient CSF flow parameters for all the patients were acquired in the chronic stage and then compared with those of the controls. CSF flow parameter differences among the three groups are shown in Figure 2. The AF (*P* = 0.039) of the patients without shunts was significantly higher than that of the controls. However, there was no significant AF difference between the patients with shunts and the controls. The PPV (*P* = 0.017), PNV (*P* = 0.007), and APF (*P* = 0.015) of the patients with shunts were significantly lower than those of the controls. There were no significant differences, however, between the patients without shunts and the controls for these parameters.

### 3.4. Relationship between Initial Clinical Data and CSF Flow Parameters

Due to dynamic changes in CSF caused by the ventriculoperitoneal shunts, we only analyzed patients without shunts for the relationships between the initial clinical data and the CSF parameters. This was done to avoid the influence of ventriculoperitoneal shunt intervention. The higher PPV (*r* = 0.974, *P* = 0.026) and PNV (*r* = 0.983, *P* = 0.017) were positively associated with a higher Evans ratio. The higher APF (*r* = −0.984, *P* = 0.016) and ANF (*r* = −0.964, *P* = 0.036) were negatively associated with a lower initial glucose level. The higher APF (*r* = −0.964, *P* = 0.036) and ANF (*r* = −0.984, *P* = 0.016) were also negatively associated with a lower maximum CSF glucose level in the acute stage.

### 3.5. Relationship between CSF Flow Parameters and Neuropsychological Function

All patients and controls were included in the partial correlation analysis to assess the relationship between CSF parameters and neuropsychological function. Higher AF was negatively associated with poorer picture arrangement test results (*r* = −0.286, *P* = 0.040) on the Wechsler Adult Intelligence Scale-III, as well as poorer short-term memory (*r* = −0.306, *P* = 0.027) and draw (*r* = −0.284, *P* = 0.041) results on the Cognitive Ability Screening Instrument. Higher ANF was negatively associated with poorer short-term memory results (*r* = −0.278, *P* = 0.046) for the Cognitive Ability Screening Instrument.

## 4. Discussion

Cryptococcal meningoencephalitis is the most frequently encountered manifestation of cryptococcal infection. The most common symptoms are headache (89%), fever (56%), personality change (17%), and limb weakness (11%), all of which are compatible with brain imaging findings. In this study, the patients with shunts had mostly poorer neuropsychological test scores than the patients without shunts, a finding which was consistent with the initial imaging findings. This may have been caused, however, by confounding factors of disease severity, including hospitalization (*z* = −2.398, *P* = 0.016) and both initial (*z* = −2.197, *P* = 0.028) and maximum (*z* = −2.064, *P* = 0.039) CSF lactate concentrations; results for all three of these variables were significantly poorer in the patients with shunts than in the patients without shunts. These findings suggest that long-term sequelae are, in some respects, established during the initial phase of the disease insofar as patients do not recover from initial problems over time, even with early ventriculoperitoneal shunt placement.

The cine MRI is a highly reproducible [15] and reliable method for taking flow measurements, whether it is performed* in vivo* [16] or* in vitro* [17]. Wide applications of cine MRI have been reported for arachnoid cysts, syringomyelia, Chiari 1 malformation, neuroendoscopic third ventriculostomy, ventriculoperitoneal shunt evaluation, and normal pressure hydrocephalus [18]. Our study results showed that the AF in the control subjects was 25.6 ± 12.9 *μ*L/beat, consistent with previous reports [19, 20].

**Figure 3:**
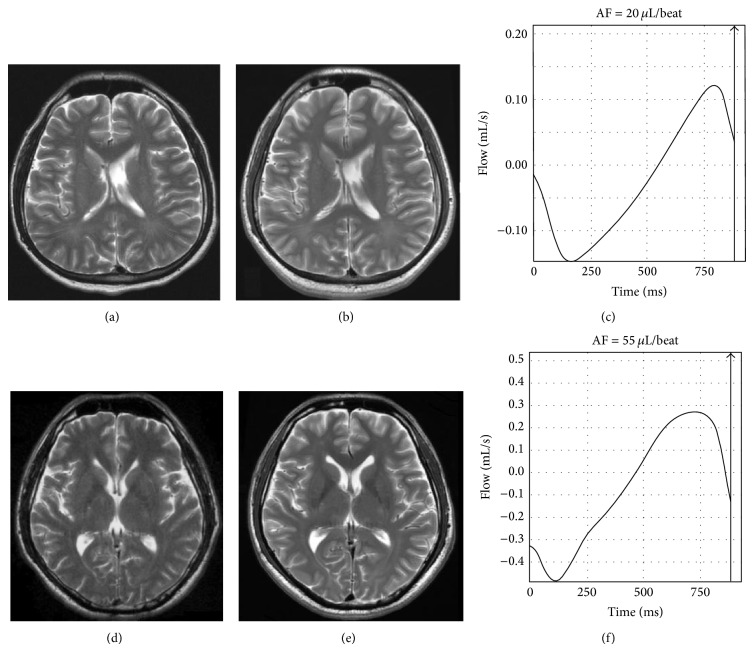
The MR imaging in (a) the acute stage and (b) at follow-up for a 27-year-old male who received a shunt and had a follow-up duration of 98 months showed no interval changes in ventricular size. (c) CSF flow patterns at follow-up showed normal wave form but a smaller longitudinal scale. The MR imaging in (d) the acute stage and (e) at follow-up for a 45-year-old male without a shunt procedure and with a follow-up duration of 97 months showed a mildly increased ventricular size. His CSF flow pattern (f) shows a more extremely increased AF than that of either the controls or the patients without shunts.

The patients without shunts had significantly higher AF than the control subjects, suggesting the persistence of chronic hydrocephalus even at the long-term follow-up (Figure 3). Meanwhile, the patients with shunts had significantly lower PPV, PNV, and APF than the controls, resulting in a similar CSF flow wave form, but on a much smaller scale than that of the patients without shunts (Figure 3). Shunt pathways appear to play important roles in dynamic changes of CSF flow. There were also correlations between initial lab data, CSF flow parameters, and neuropsychological scores, suggesting that CSF flow parameters gathered by cine MRI may be associated with long-term neuropsychological sequelae and correspond to initial disease severity.

The causes of chronic hydrocephalus in cryptococcal meningoencephalitis patients remain unclear; however, some clues in the changes of CSF dynamics may be helpful for finding answers. The elevated AF in patients without shunts indicates chronic hydrocephalus after long-term follow-up. In addition to CSF malabsorption, decreased intracranial compliance may lead to restricted arterial pulsations and increased capillary pulsations [21], thereby causing chronic hydrocephalus. The CSF flow dynamics are usually classified as hypermotile/hyperdynamic, normal, or hypomotile/hypodynamic according to CSF flow parameters in cine MRI [18, 22]. Typical hypomotile CSF flow diseases are Chiari I malformation [23] and aqueductal stenosis [24], with decreased average flow. In contrast, the chronic phase of cryptococcal meningoencephalitis, during which patients without shunts exhibit increased AF and patients with shunts exhibit normal AF, suggests totally different CSF flow dynamics.

In the patients without shunts, the AF was 39.013 ± 13.699 *μ*L/beat, which was significantly higher (*f* = 3.516, *P* = 0.038) than the AF for the controls (25.6 ± 12.9 *μ*L/beat). A similar result has been noted in patients with normal pressure hydrocephalus, which is a well-known disease with hypermotile CSF flow [25]. These patients have decreased intracranial compliance and, thus, present with hypermotile CSF flow [26].

In the patients without shunts, the increased AF was accomplished with both decreased PPV and PNV. Similar findings have also been observed in normal pressure hydrocephalus patients without shunts. In the early stage of communicating hydrocephalus, the same brain mass expands inward, compressing the ventricle and causing much greater outflow volume and flow velocity [27]. In the chronic phase, brain atrophy with parenchymal tissue loss may result in decreased arterial inflow during systole, the primary force behind the CSF pump, causing a subsequent decrease in aqueduct CSF flow velocity [27]. In normal pressure hydrocephalus, progressive AF reduction with worsening clinical symptoms is a sign of progressive cerebral ischemic injury rendering normal pressure hydrocephalus irreversible [28]. The findings of increased AF with decreased PPV and PNV in cryptococcal meningoencephalitis patients without shunts similarly imply persistent or irreversible inflammatory processes and brain atrophy.

In the patients with shunts, normal AF and lower PPV and PNV findings are consistent with a previous study on communicating hydrocephalus after placement of ventriculoperitoneal shunts. These results suggest that CSF flow is effectively shifted to the pathway created by the ventriculoperitoneal shunt and that a normal AF in the aqueduct is maintained by adjusting the peak velocities [29]. Early intervention has been proven to stop hydrocephalus in normal and cryptococcal meningoencephalitis, providing a favorable prognosis [30]. However, the patients with shunts received lower scores on most tests than the patients without shunts. These results suggest that most neuropsychological sequelae occur in the acute stage according to disease severity and are irreversible, despite the continuous normal CSF dynamics pattern.

Patients with bacterial meningitis often suffer from neurologic and neuropsychological sequelae [31, 32]. The impaired cognitive functioning of patients with cryptococcal meningoencephalitis, especially with regard to short-term and working memory, is also observed in patients with bacterial and viral meningitis [33]. Interestingly, in the block design, orientation, and short-term memory tests, the patients with shunts had higher scores than the patients without shunts, in spite of the fact that they also had more severe initial presentations. This implies that ventriculoperitoneal shunts not only save patients' lives but also protect patients from certain specific aspects of cognitive function injury. Similar results have also been reported for idiopathic normal pressure hydrocephalus patients, with both “frontal” executive functions and “posterior cortical” functions being preserved after shunt surgery [34].

A cryptococcal meningoencephalitis patient without a ventriculoperitoneal shunt usually has a better prognosis in the acute infective stage. However, explorations of the correlations between bacterial burden from CSF analysis and long-term CSF dynamics have found that a lower glucose concentration and higher Evans ratio in the acute stage are associated with chronic hypermotile CSF dynamics. Worse results on neuropsychological tests, including picture arrangement and short-term memory, can be predicted by CSF parameters via cine MRI. Further validation of cine MRI for both the early prediction of long-term outcomes and the monitoring of meningitis treatment responses should be conducted.

This retrospective study has some limitations. First, cryptococcal meningoencephalitis is a rare disease for a person with normal immunity. Thus, only 18 HIV-seronegative patients were included in this study. As a result, both the disease follow-up duration and the initial image modality were difficult to control. Second, gaps still exist between the causal relationships of chronic hydrocephalus and cryptococcal meningoencephalitis pathogenesis. The study did not answer whether or not cognitive impairment resulted from the bacterial burden alone or from the stress pressure caused by hydrocephalus; both may result in white matter changes that can alter cognitive function. Finally, it is impossible to examine the effects of preexisting major psychiatric illnesses and their corresponding anatomic defects that result in cognition deficits.

## 5. Conclusion

In HIV-seronegative cryptococcal meningoencephalitis, cine MRI may be used to detect differences in CSF flow parameters between chronic disease stage patients with and without ventriculoperitoneal shunts. In the present study, these CSF flow parameters were correlated with the acute stage disease severity and chronic stage neuropsychological results. Using cine MRI for acquiring CSF parameters can help determine the presence of chronic hydrocephalus in cryptococcal meningoencephalitis patients. It may also determine the relationship between CSF parameters and neuropsychological sequelae.

## Figures and Tables

**Table 1:** Demographic data of cryptococcal meningitis patients and controls.

Demographics	Patients with shunts	Patients without shunts	Normal	*f* or *z*	*P*
Gender (male/female)	9/1	7/1	28/4	0.022	0.978
Age at follow-up	53.70 ± 14.30	54.00 ± 9.87	51.47 ± 13.30	0.190	0.827
Education	9.30 ± 5.46	10.63 ± 4.63	12.56 ± 4.22	2.184	0.124
Duration of follow-up (months)^a^	73.5 (21.25, 113.25)	116 (38.5, 130.75)	—	−1.289	0.197
Hospitalization duration (days)^a^	53 (33.5, 60.5)	25 (17, 43)	—	−2.398	**0.016**
Glasgow Coma Scale at admission^a^	15 (13.75, 15)	15 (14, 15)	—	−0.304	0.761
Glasgow Coma Scale at discharge^a^	15 (13.75, 15)	15 (15, 15)	—	−0.853	0.394
CSF Cryptococcal Ag titer at admission^a^	1024 (320, 1536)	384 (96.5, 2816)	—	−0.842	0.400
Max CSF Cryptococcal Ag titer during hospitalization^a^	1024 (320, 2560)	1024 (96.5, 2816)	—	−0.311	0.756
CSF white cell count (/mm^3^)^a^	10 (43.75, 400.5)	152 (40.5, 290)	—	−0.245	0.806
CSF protein at admission (g/L)^a^	201.5 (96.75, 355.43)	156 (91.6, 189)	—	−1.073	0.283
CSF protein max during hospitalization (g/L)^a^	210.4 (130.5, 504)	169.5 (91.6, 317)	—	−1.429	0.153
CSF lactate at admission (mmol/L)^a^	28.6 (22.85, 52.175)	18.1 (9.1, 24)	—	−2.197	**0.028**
CSF lactate max during hospitalization (mmol/L)^a^	41.7 (29.9, 75.4)	24 (20.1, 51.8)	—	−2.064	**0.039**
CSF glucose at admission (mmol/L)^a^	45.5 (18.25, 58.75)	36 (22, 49)	—	−0.976	0.329
CSF glucose max during hospitalization (mmol/L)^a^	48 (30.5, 125.5)	53 (25, 70)	—	−0.582	0.560
Serum sodium (mmol/L)	138.90 ± 3.76	137.86 ± 2.55	—	0.446	0.516
Creatinine level at discharge (*μ*mol/L)	1.0867 ± 0.27	1.22 ± 0.33	—	1.125	0.312
Evan ratio in admission	27.11 ± 3.04	27.96 ± 5.34	—	0.065	0.802
Evan ratio in follow-up	27.13 ± 2.86	26.12 ± 2.52	—	0.877	0.365

^a^Nonparametric independent samples test was used for the non-Gaussian distribution of data. Bold-faced values represent significant differences between groups (*P* ≤ 0.05).

**Table 2:** Neuroimaging findings of the patients in the acute stage and at follow-up.

Patient number	Antigen titer	Acute MRI finding	MRI f/u finding	Image f/u interval (months)
Patients with shunts				
1	1 : 1024	Meningeal enhancement, mild Hyperintensity over thalamus	Meningeal enhancement, progression Hyperintensity over bilateral frontal and cerebellum	4
2	1 : 1024+	Meningeal enhancement, diffuse	Dilated lateral left frontal ventricle	98
3	1 : 512	Meningeal enhancement, diffuse VR dilatation, right side Hyperintensity over right BG	Meningeal enhancement, regressive change Hyperintensity over bilateral frontoparietal, basal ganglia, and thalami	24
4	1 : 1024	Meningeal enhancement, diffuse Hyperintensity over bilateral occipital, right high frontal, and parietal, right genus of corpus callosum	Meningeal enhancement, diffuse Hyperintensity over right genus of corpus callosum and bilateral occipital lesion regression	13
5	Negative	Dilated bilateral ventricles (CT)	Hyperintensities over bilateral frontoparietal area	124
6	>1 : 1024	Meningeal enhancement, diffuse Hyperintensity over suprasellar area	Complete regression	50
7	1 : 128	Meningeal enhancement, diffuse Hyperintensity over bilateral cerebellum and bilateral BG	Dilated lateral ventricles Hyperintensity over bilateral cerebellum	35
8	Negative	Prominent ventricles Hyperintensity over bilateral BG	Complete regression	111
9	1 : 8	Meningeal enhancement, diffuse VR dilatation, right side	Meningeal enhancement, diffuse VR dilatation, right side	120
10	1 : 2048	Hyperintensity over left frontoparietal area Dilated bilateral ventricles	Complete regression	97

Patients without shunts				
11	1 : 8+	VR dilatation, bilateral	Complete regression	117
12	1 : 8	No active lesion	No active lesion	132
13	1 : 256+	No active lesion	No active lesion	97
14	Negative	No active lesion (CT)	No active lesion	115
15	1 : 128	Meningeal enhancement over posterior fossa and perivascular space of supratentorium, VR dilatation, bilateral	Complete regression	12
16	None	No active lesion (CT)	No active lesion	127
17	1 : 256	Meningeal enhancement over right occipital and bilateral central sulci Hyperintensity over right occipital and bilateral parietal area	Dilated lateral ventricles	19
18	Negative	No active lesion (CT)	Dilated lateral ventricles	168

**Table 3:** Neuropsychological rating scores among groups.

Neuropsychological tests	All patients	Patients with shunts	Patients without shunts	Normal	*F*_1_	*P*_1_	*F*_2_	*P*_2_
Wechsler Adult Intelligence Scale-III								
Information	9.06 ± 2.46	9.33 ± 2.00	8.75 ± 3.01	11.18 ± 2.85	2.810	0.101	2.239	0.119
Vocabulary	9.47 ± 3.36^#^	8.44 ± 3.21^§^	10.62 ± 2.82	12.06 ± 2.79^#§^	4.057	**0.050**	3.131	0.054
Similarity	8.41 ± 3.69^#^	7.55 ± 3.04^§^	9.37 ± 4.30	11.50 ± 2.03^#§^	10.059	**0.003**	6.153	**0.004**
Comprehension	8.94 ± 3.83^#^	8.33 ± 3.50	9.62 ± 4.30	11.96 ± 3.08^#^	4.946	**0.031**	2.618	0.085
Arithmetic	9.41 ± 3.14	9.33 ± 2.91	9.50 ± 3.58	10.96 ± 2.49	1.785	0.188	0.875	0.424
Digit span	8.71 ± 3.16^#^	8.33 ± 3.96	9.12 ± 2.10	12.28 ± 3.64^#^	7.019	**0.011**	3.471	**0.040**
Letter-number sequencing	7.88 ± 4.61	8.11 ± 4.22	7.62 ± 5.23	10.68 ± 3.55	3.156	0.085	1.606	0.216
Picture complete	8.06 ± 2.66^#^	7.66 ± 2.00	8.50 ± 3.33	10.53 ± 2.89^#^	6.549	**0.014**	3.481	**0.040**
Block design	8.47 ± 3.83^#^	8.77 ± 3.52	8.12 ± 4.35^*£*^	11.90 ± 3.10^#*£*^	9.280	**0.004**	4.772	**0.013**
Matrix reasoning	8.94 ± 2.97	9.00 ± 2.64	8.87 ± 3.48	10.81 ± 3.51	1.085	0.303	0.594	0.556
Digit symbol coding	8.06 ± 3.58^#^	7.55 ± 2.50^§^	8.62 ± 4.62	11.53 ± 2.67^#§^	9.507	**0.004**	4.809	**0.013**
Picture arrangement	8.71 ± 3.31	9.11 ± 3.14	8.25 ± 3.65	10.65 ± 2.96	1.709	0.198	1.262	0.293

Cognitive Ability Screening Instrument								
Attention	7.23 ± 1.09	6.77 ± 1.37^§&^	7.75 ± 0.46^&^	7.84 ± 0.44^§^	3.870	0.055	6.475	**0.003**
Orientation	15.88 ± 3.69^#^	16.55 ± 3.24	15.12 ± 4.22^*£*^	17.71 ± 0.77^#*£*^	4.076	**0.050**	3.413	**0.042**
Abstract thinking	9.35 ± 2.32^#^	8.88 ± 2.20	9.87 ± 2.47	10.78 ± 1.26^#^	4.560	**0.038**	2.889	0.067
Mental Manipulation	8.41 ± 1.80^#^	8.44 ± 1.42	8.37 ± 2.26	9.31 ± 1.17^#^	4.174	**0.047**	2.042	0.142
Short-term memory	8.48 ± 3.33^#^	8.85 ± 3.36	8.06 ± 3.47^*£*^	10.71 ± 1.76^#*£*^	5.49	**0.024**	3.376	**0.043**
Long-term memory	9.65 ± 0.79	9.77 ± 0.66	9.50 ± 0.92	9.93 ± 0.35	1.426	0.239	1.606	0.212
Language	9.73 ± 0.50	9.60 ± 0.59	9.87 ± 0.35	9.85 ± 0.54	0.506	0.481	1.308	0.281
Semantic fluency	6.94 ± 2.75^#^	6.66 ± 3.20	7.25 ± 2.31	8.93 ± 1.47^#^	5.835	**0.020**	2.869	0.068
Drawing	9.06 ± 1.60	8.77 ± 1.78	9.37 ± 1.40	9.81 ± 0.592	3.828	0.057	2.606	0.085
Total score	84.74 ± 14.51^#^	84.34 ± 14.73	85.18 ± 15.24	94.91 ± 4.74^#^	8.945	**0.005**	4.377	**0.019**

*F*_1_ and *P*_1_ represent comparison between all patients and the controls.

*F*_2_ and *P*_2_ represent comparison among the patients with/without shunts and controls.

^#^Significant difference between all patients and the controls.

^§^Significant difference between the patients with shunts and controls.

^*£*^Significant difference between the patients without shunts and controls.

^&^Significant difference between the patients with and without shunts.

Bold-faced values represent significant differences (*P* ≤ 0.05).

## Abbreviations

AF: Average flow
ANF: Average negative flow
APF: Average positive flow
PNV: Peak negative velocity
PPV: Peak positive velocity.
